# Value of ultrasonography parameters in diagnosing polycystic ovary syndrome

**DOI:** 10.1515/med-2022-0505

**Published:** 2022-06-20

**Authors:** Augustina Gyliene, Vestina Straksyte, Inga Zaboriene

**Affiliations:** Medical Academy, Lithuanian University of Health Sciences, Kaunas, Lithuania; Department of Radiology, Lithuanian University of Health Sciences, Eivenių Str. 2, Kaunas, Lithuania; Department of Radiology, Lithuanian University of Health Sciences, Kaunas, Lithuania

**Keywords:** polycystic ovary syndrome, ovarian volume, ultrasonography, Doppler, follicle number

## Abstract

Polycystic ovary syndrome (PCOS) is a common endocrinopathy among women of reproductive age associated with hyperandrogenism, oligo-amenorrhea, and infertility. Symptoms and their severity vary among the individuals. If the manifestation is mild, PCOS may remain undiagnosed. In more severe cases, it results in a spectrum of symptoms of metabolic syndrome, insulin resistance, and cardiovascular diseases. The diagnosis is established after a physical examination and evaluating the patient’s hormonal profile. In addition to these required methods, ultrasonographic assessment of the patient’s ovaries is another non-invasive, cheap, and time-saving tool, making the examination more profound and leading to the correct diagnosis. Specific ultrasonographic parameters are used to tell the healthy and polycystic ovaries apart: the ovarian volume (OV), ovarian follicle count, follicle distribution pattern, ovarian stromal echogenicity, and the resistance and pulsatility indices assessed using the Doppler function. This review evaluated the selected articles and ascertained the ultrasonographic parameters that accurately predict PCOS. This systematic review showed that the most valuable ultrasonographic parameters in diagnosing PCOS are the OV and follicle number per ovary.

## 1 Introduction

Polycystic ovary syndrome (PCOS), or Leventhal and Stein disease, is an endocrine and metabolic disorder that most commonly affects women of reproductive age. Most European women diagnosed with PCOS are 35–44 years old [1]. The prevalence rate of this disorder among adolescents is also concerning (97.83 per 100,000) [1]. PCOS manifests with a broad diversity of clinical symptoms associated with hyperandrogenemia and insulin resistance (IR). Evidence confirmed a key role for IR and compensatory hyperinsulinemia in the pathogenesis of PCOS, which may be exacerbated by concomitant obesity, which affects approximately 50% of women with PCOS (occurring in about 80% of obese women with PCOS and 30–40% of lean women) [2,3,4]. IR has been consistently observed among many women with PCOS, but this is excluded from any diagnostic criterion.

The syndrome is often related to severe conditions such as diabetes mellitus, cardiovascular diseases, and metabolic syndrome (MetS) [5,6,7].

It is crucial to establish a proper diagnosis and treat PCOS before it causes severe or even life-threatening problems. However, it is not always an easy task. Treatment options include lifestyle changes, medicines (isoforms of inositol), or surgical methods [4,8].

Over the years, the diagnostic criteria for PCOS have been changing. The initial diagnostic criteria were established at the National Institutes of Health consensus conference. These criteria were broadened several years after describing four main PCOS phenotypes [9]. Two main features are required to diagnose PCOS: the presence of hyperandrogenism and chronic oligo-anovulation if no other disorders cause these conditions [10]. The other criteria were introduced at the conference held in Rotterdam, the Netherlands [9]. Consequently, specific ultrasound features for ovarian morphology were added to the two existing criteria, thus expanding the definition of PCOS. An ovary was considered polycystic if the ovarian volume (OV) was greater than 10 cm^3^ and/or the number of follicles (FNPO) measuring 2–9 mm was 12 per ovary or greater [11].

Various publications express the significance of these ultrasound parameters in establishing PCOS diagnosis. The current article assesses the value of ultrasound parameters in diagnosing PCOS.

## 2 Evaluation of criteria for PCOS

To date, the expanded Rotterdam criteria are widely accepted and recommended using international evidence-based guidelines [12].

Ultrasound features for polycystic ovary morphology have slightly changed – follicle number per ovary was altered to 20 or more (Table 1) [12]. Many publications have recently proved that ultrasound is valuable in doubtful cases, especially with hormonal assays [13,14,15].

**Table 1:** Main ultrasound parameters

Ultrasound features	
FNPO	≥12 per ovary* measuring 2–9 mm
	≥20 per ovary** measuring 2–9 mm
OV	**>**10 mL, Ensuring no corpora lutea, cysts, or dominant follicles are present
FDP	Predominantly peripheral

* – Rotterdam criteria, ** – International evidence-based guideline for the assessment and management of polycystic ovary syndrome 2018 [12].

Despite the OV and FNPO, a few more ultrasound features help diagnose PCOS: follicle distribution pattern (FDP), antral follicle count (AFC), resistance index (RI), pulsatility index (PI) of uterine and ovarian arteries, and ovarian stromal echogenicity [15,16].

## 3 Materials and methods

The systematic article search was performed according to PRISMA guidelines. The Google Scholar and MEDLINE (PubMed) electronic databases were searched. The terms “polycystic ovary syndrome,” “PCOS,” “ultrasound,” “Doppler,” “ovarian volume,” “antral follicle count,” “ovarian stroma” were used in the process. Only articles written in the English language and published between the years 2014 and 2021 were included. The initial search resulted in 8,062 articles in both databases. After removing the duplicates and all systematic reviews, meta-analyses, case reports, and animal studies, the article titles or abstracts were reviewed manually, and the irrelevant article titles or abstracts were excluded. The studies were considered relevant if they used the ultrasound technique, the sample size was larger than 30 participants, and the PCOS was their main study condition. The advanced search resulted in 12 articles enrolled in the review [17,18,19,20,21,22,23,24,25,26,27,28].

## 4 Results

Twelve studies used ultrasound to evaluate women with PCOS symptoms. Nine authors conducted case–control studies. Three authors organized cross-sectional studies. The main features of the studies are presented in Table 2.

**Table 2:** Characteristics of the studies that used ultrasound to evaluate PCOS

Study, year	PCOS/Controls (*n*)	Mean BMI (kg/m^2^) PCOS/Controls	Age range	Mean age	Transducer frequency (MHz)	Menstrual cycle day	Doppler feature usage
Ali et al., 2016 [17]	90/90	−/−	16–38	27	2–7.2	3–7	−
Chawla and Anand, 2020 [18]	35/35	−/−	<35	−	3–9	3–5	+
Manzoor et al., 2019 [21]	50/50	−/−	PCOS 13–42/Controls 14–45	PCOS 23/Controls 27	2–7	−	+
Anum et al., 2019 [22]	69/69	−/−	20–45	27.65	3–7.5	6–14	+
Ozdemir et al., 2015 [23]	42/38	24.4/22.5	−	PCOS 22.3/Controls 22.7	6.5	2–5	+
Christ et al., 2015 [24]	49/−	33.3/−	19–36	27.5	9	−	−
Bano and Tariq [25]	120/−	−/−	20–37	24.3	−	8–16	+
Dwivedi et al., 2019 [26]	100/100	26.27/22.84	−	26.2	3.5–5	3–5	+
Sipahi et al., 2019 [27]	15/81	31.8/24.3	−	PCOS 25.8/Controls 23.3	1–8	1–7	+
Jarrett et al., 2019 [28]	87/67	32.0/23.6	18–38	PCOS 27/Controls: 27	5–9 or 6–12	−	−
Younesi et al., 2019 [19]	45/32	28.1/24.3	−	PCOS 28.1/Controls 32	−	2–5	+
Ahmed et al., 2020 [20]	25*; 25**/25^X^; 25^XX^	29.37*; 21.48**/28.19^X^; 22.77^XX^	20–40	31	2–5 and 7.5	3–7	−

Abbreviations: BMI – body mass index; PCOS – polycystic ovary syndrome; *PCOS with obesity; **PCOS-only; ^x^ – obesity-only; ^xx^ – controls.

+: Doppler was used.

−: Doppler wasn't used.

### 4.1 OV

Nine articles assessed the importance of OV in diagnosing PCOS and predicting the severity of related conditions [17,18,19,20,23,24,26,27,28]. The highest value of the mean OV in the PCOS group among the studies was 16.25 mL. The lowest value was 9.65 mL. In the control groups, the mean value of OV varied between 4.86 and 9.6 mL. The mean OV values are presented in Table 3.

**Table 3:** Mean OV, FNPO, and AFC

Study, year	Mean OV (mL) PCOS/Controls	*P* value	Mean FNPO PCOS/Controls	*P* value	Mean AFC PCOS/Controls	*P* value
Ali et al., 2016 [17]	9.65/9.3	—	–/–	—	–/–	—
Chawla and Anand [18]	15.72/4.93	<0.01	17.39/5.72	<0.01	–/–	—
Ozdemir et al., 2015 [23]	11.43/4.86	<0.05	–/–	—	–/–	—
Christ et al., 2015 [24]	14/–	—	–/–	—	77/–	—
Dwivedi et al., 2019 [26]	16.25/5.5	<0.0001	14.39/3	<0.0001	–/–	—
Sipahi et al., 2019 [27]	11.7/9.6	0.027	–/–	—	32.3/29.6	>0.05
Jarrett et al., 2019 [28]	11/7	<0.05	45/25	<0.05	–/–	—
Younesi et al., 2019 [19]	16/8.1	<0.01	18.3/7.1	≤0.05	–/–	—
Ahmed et al., 2020 [20]	11.1* and 11.2**/9.4^x^; 9.1^xx^	<0.05	–/–	—	–/–	—

Abbreviations: AFC** – **antral follicle count; FNPO** – **follicle number per ovary; OV** – **ovarian volume; PCOS** – **polycystic ovary syndrome; *PCOS with obesity; **PCOS-only; ^**x**^ – obesity-only; ^**xx**^ **– **controls.

–: Data are not available.

According to the recommendations of the newest evidence-based guidelines, an ovary is enlarged when its volume is greater than 10 cm^3^ [12]. Although high OV is thought to be a reliable PCOS marker, not all authors managed to prove its superiority to other ultrasound features in diagnosing this disease. Ali et al. found no statistically significant difference between patients with PCOS’ OV and the healthy women’s OV. Only 16.6% of the evaluated ovaries were above the normal volume range [17]. Christ et al. showed that the mean OV of the patients was elevated to 14 mL. However, no statistically significant links between OV and reproductive dysfunction were found [24].

In contrast, Sipahi et al. proved that OV is a valuable ultrasound feature helpful in predicting the occurrence of MetS in patients with PCOS [27]. The authors found that the mean OV of the PCOS and MetS patient group was significantly higher than in PCOS-only group [27]. A study by Jarrett et al. investigated the difference between the right and left ovaries in patients with PCOS and controls and found that the right ovary was more prominent in both groups [28]. Also, mean OV was significantly higher in the PCOS group than in the control [28]. It was concluded that the prevalence of developing MetS increases in patients with PCOS with larger ovaries.

Ahmed et al. studied four groups of participants: obese patients with PCOS, non-obese patients with PCOS, obese women without PCOS, and healthy controls. The mean OV was highest in the PCOS-only group (11.2 mL) and lowest in the healthy control group (9.1 mL) [20]. A similar study by Younesi et al. found that the mean OV was highest in the PCOS group; however, in contrast to Jarret et al., the left ovary was more prominent in all groups [22].

Additionally, the mean OV was significantly higher in obese participants than in non-obese ones [20]. Chawla and Anand and Dwivedi et al. proved that the OV is considerably higher in patients with PCOS than in healthy individuals [18,26]. Chawla and Anand found that the mean OV in the PCOS patient group was significantly higher than in the control group [18]. Dwivedi et al. obtained similar results [26].

### 4.2 FNPO and AFC

Chawla and Anand and Dwivedi et al. compared the FNPO in PCOS and control groups and obtained statistically significant results [18,26]. Both studies involved only follicles that measured 2–9 mm. Younesi et al. tested patients with PCOS, women with PCO morphology, and a healthy control group [19]. The mean FNPO was significantly higher among patients with PCOS than healthy women [19]. All mean FNPO and AFC values are presented in Table 3.

Jarrett et al. investigated the differences in FNPO between the right and left ovaries with PCOS and controls [28]. The researchers found that the FNPO was almost two times higher in both ovaries of the PCOS cohort than it was among the controls. Moreover, 94% of patients with PCOS had the FNPO higher than 20, and 89% had it higher than 25 in both ovaries [28]. The authors concluded that FNPO has tremendous diagnostic potential for PCOS.

Christ et al. and Sipahi et al. attempted to find correlations between the AFC and different PCOS manifestations [24,27]. The study by Christ et al. assessed the clinical, ultrasonographic, and hormonal features of patients with PCOS to find associations between them [24]. They discovered that the mean AFC of 49 patients with PCOS was 77, ranging from 36 to 145. The AFC was positively related to free testosterone and androstenedione levels and the LH:FSH ratio. In another study by Sipahi et al., the difference in AFC between PCOS and PCOS with MetS patient groups was not significant (*P* > 0.05) [27]. The mean AFC of the PCOS-only patient group was 29.6; in the group of PCOS with MetS, it was slightly higher** **–** **32.3.

### 4.3 FDP

Ali et al. found that the classic FDP of PCOS, the ultrasonographic “string of pearls” sign, appeared in most patients in the case group (91.1%). Other patients (8.8%) showed normal morphology, with follicles equally distributed within the ovarian stroma [17]. FDP was normal in all 90 controls. Younesi et al. also discovered some dissimilarities in FDP among the three participant groups. Although no significant difference was observed between patients with PCOS and women with PCO morphology (82.2 and 80.6% women with peripheral distribution of follicles, respectively), these groups showed a significant difference from the control group, in which the “string of pearls” sign appeared in 46.8% of participants [19]. Christ et al. investigated the reproductive and metabolic features of patients with PCOS. They assessed the correlations between them and the FDP, but there was no statistically significant link between them [24]. Sipahi et al. did not find a notable difference between the FDP of PCOS-only patients and patients with PCOS with coexisting MetS (1.7 and 1.8, respectively) [27].

### 4.4 Ovarian stromal echogenicity

Ovarian stromal echogenicity is an important PCOS marker. The study by Dwivedi et al. proved a definite difference between healthy women and patients with PCOS’s ovarian stromal echogenicity [26]. Hyperechogenic stroma was seen in 98% of patients with PCOS and 4% of healthy women. Chawla and Anand also discovered a statistically significant difference in stromal echogenicity between the controls and patients with PCOS [18]. Only 7.1% of 35 healthy women had hyperechoic ovarian stroma, and among 35 patients, this feature occurred in 60% of cases.

### 4.5 RI and PI of uterine and ovarian arteries

Increased blood flow in the ovarian stroma is a critical PCOS feature, occurring due to the hyperactive angiogenesis in the ovary. The abundant blood flow may disrupt normal folliculogenesis, stimulating the growth of multiple primary follicles instead of a few leading ones [16,29]. The ultrasonographic Doppler feature is used to detect the increased blood flow by measuring the PI and RI of the ovarian stromal or uterine arteries [16]. The mean RI of the uterine artery ranged between 0.877 and 0.96 in the PCOS and the control group between 0.868 and 0.92. The RI of ovarian stromal arteries in the PCOS group was from 0.45 to 0.83, and in the control group between 0.50 and 0.84. The mean PI of the uterine artery was from 3.04 to 3.89 in the PCOS group and 2.2 in the control group. The PI values of the ovarian stromal arteries varied between 0.815 and 2.5 in the case group and 1.3 and 4.2 in the control group. All RI and PI mean values are presented in Table 4.

**Table 4:** Mean values of RI and PI in uterine and ovarian stromal arteries

Study, year	PCOS/Controls (*n*)	Mean RI PCOS/Controls (*n*)		Mean PI PCOS/Controls (*n*)		*P* value
		Uterine artery	Ovarian stromal arteries	Uterine artery	Ovarian stromal arteries	
Chawla and Anand, 2020 [18]	35/35	0.91/0.92	0.515/0.675	3.04/2.2	0.815/1.35	<0.01
Manzoor et al., 2019 [21]	50/50	–/–	0.6/0.79	–/–	1.24/2.16	—
Sarwar et al., 2019 [22]	69/69	R–0.886/0.879; L–0.877/0.868	–/–	–/–	–/–	>0.05
Ozdemir et al., 2015 [23]	42/38	–/–	0.45/0.84	–/–	0.89/1.3	<0.05
Bano and Tariq [25]	120/–	0.93/–	–/–	3.89/–	–/–	—
Dwivedi et al., 2019 [26]	100/100	–/–	0.52/0.71	–/–	1.15/4.2	<0.0001
Sipahi et al., 2019 [27]	15* 81**/–	–/–	0.81* 0.83**/–	–/–	2.2 2.5/–	>0.05
Younesi et al., 2018 [19]	45/32	0.96/0.87	0.50/0.50	–/–	–/–	>0.05

Abbreviations: L** – **left; PI** – **pulsatility index; R** – **right; RI** – **resistance index; *with metabolic syndrome; **without metabolic syndrome.

– Data are not available.

## 5 Discussion

PCOS is a highly prevalent condition that affects 1 of 10 women of reproductive age worldwide [30]. The PCO morphology is defined by various ultrasonographic features that, along with hormonal assays, help the physician diagnose PCOS. PCOS is composed of many related conditions. No “gold standard” diagnostic test can predict it alone. However, the ultrasonographic evaluation is essential in establishing or solidifying the diagnosis [30,31].

Testing for PCOS in early puberty and adolescence is still an issue, and the ultrasonographic evaluation might be controversial. The syndrome might affect women at the beginning of their reproductive years. In this case, transvaginal ultrasonography is considered more accurate. This method is inappropriate for children and sexually inactive adolescent girls [31]. The international guidelines recommend using ultrasound for patients more than eight years have passed after their first menstruation [12]. During puberty, the body goes through significant hormonal changes, which naturally stimulate the growth of ovarian follicles. This state appears similar to the PCO morphology [31]. Witchel et al. stated that ultrasonography is unnecessary for pediatric and adolescent patients and leads to overdiagnosis [32].

OV is a widely used ultrasonographic parameter in establishing the diagnosis of PCOS. Senaldi et al. stated that increased OV was significantly associated with circulating testosterone and insulin and IR [33]. Hyperinsulinemia and elevated serum LH are essential in ovarian enlargement and androgen synthesis in patients with PCOS [33]. Numerous studies have proved that patients with PCOS have larger ovaries than the controls [18,19,20,23,26,27,28]. However, the range of values of the mean OV is extensive, not only in patients but also in healthy women. Because healthy women have PCO morphology, their enlarged ovaries lead to PCOS overdiagnosis as one of the Rotterdam criteria [34]. This review showed a minimal difference between the lowest mean OV of the patients with PCOS and the highest mean OV value in the control groups (9.65 and 9.6 mL, respectively). Data suggest that women have normal-sized ovaries in PCOS patient groups and larger than normal in the control groups.

FNPO is another commonly used ultrasonographic parameter associated with PCOS. The number of ovarian follicles becomes markedly higher in patients with PCOS due to increased androgen and anti-Müllerian hormone levels [35]. Jarrett et al. found it superior to OV and follicle numbers per section in detecting polycystic ovaries when a high-frequency transvaginal transducer is available [28]. The cut-off value of FNPO is the most specific for PCOS. Even though the international evidence-based guidelines accepted the cut-off of 20 or more follicles per ovary, some authors indicate different FNPO thresholds [12]. Dewailly et al. suggested using a cut-off of 25 or more follicles per ovary for 18–35-year-old women. Lujan et al. found that an FNPO of 26 or more per ovary is a reliable threshold for detecting PCOS [36,37]. It is complicated to determine a single cut-off value for FNPO because physicians often use different ultrasound machines and follicle-calculating methods. Additionally, the results depend on the skills and experience of the observer.

Scientists have noticed that the widely accepted Rotterdam criteria can lead to misdiagnosing PCOS in middle-aged women. For that reason, PCOS might be underdiagnosed in older women if the currently used cut-offs of OV (>10 cm^3^) and FNPO (>20) are applied [14]. Kim et al. lowered the cut-off values of OV and FNPO for women older than 30 years because, at that age, the volume of the ovary and follicle count starts to decrease [38].

**Figure 1:**
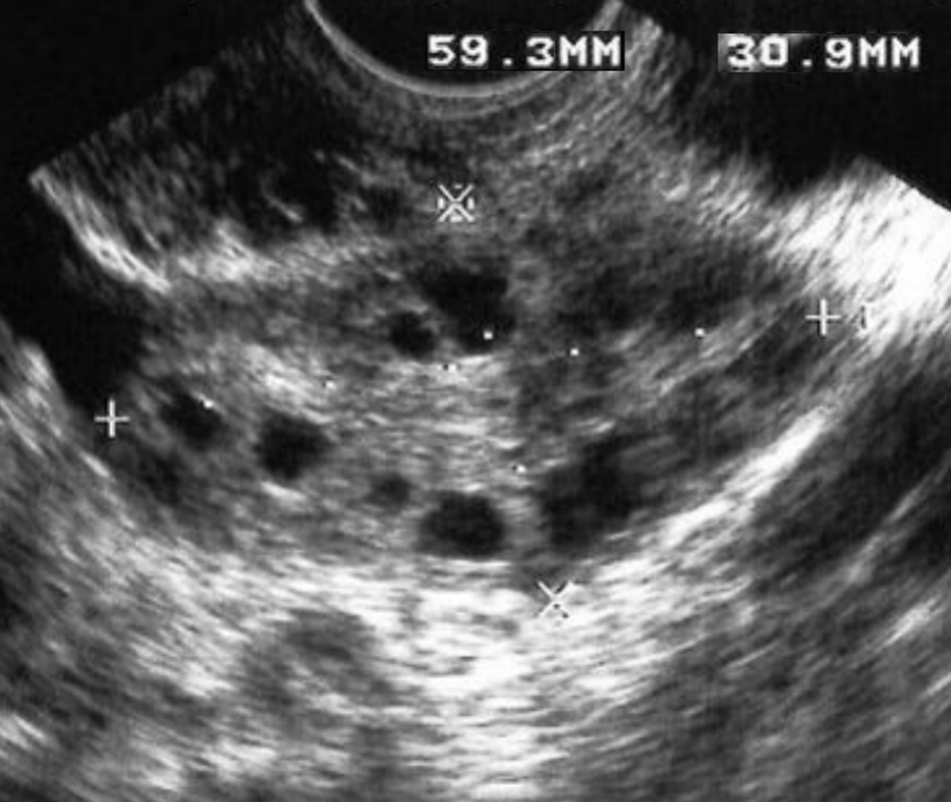
Transverse US image of the left ovary demonstrates the “string of pearls” sign.

FDP is an essential feature of PCOS. The follicles might be scattered throughout the ovary or distributed peripherally around the ovarian stroma. The first variant is considered normal, and the second is the so-called ultrasonographic “string of pearls” sign, a typical finding in PCOS (Figure 1) [39].

The ovarian stromal brightness in patients with PCOS might be related to increased serum concentrations of vascular endothelial growth factor, which encourages the formation of new vessels around the ovarian follicles and thus leads to the development of hyperechoic stroma sign [32]. The hyperechoic or brighter grayscale tone of the ovarian stroma is determined subjectively and mainly depends on the experience of the examinator. Therefore, not many authors use this ultrasonographic parameter daily [19].

The ovarian blood flow of a patient with PCOS is often abnormal. The imbalance of angiogenic and antiangiogenic factors induces vascular growth within the ovarian stroma. The stromal arteries supply blood to the small ovarian follicles because their walls do not contain any vessels [40]. When the blood flow in the stromal arteries increases, it also induces abnormal follicle growth – numerous small follicles accumulate around the ovarian stroma, their further maturation is restricted, and the failure to select dominant follicle results in anovulation [35]. The increase in ovarian blood flow also alters the risk of developing ovarian hyperstimulation syndrome. The ultrasonographic Doppler parameters, such as RI and PI, help detect abnormal ovarian blood flow and diagnose this condition early. Since the enhanced ovarian blood flow causes most PCOS symptoms, scientists revealed that vascular growth restriction is a suitable PCOS treatment method [40]. The medicines inhibit angiogenic factors, restoring normal ovarian blood flow and reducing PCOS symptoms.

Numerous studies have emphasized the association between the ultrasound parameters and clinical features of patients with PCOS. Christ et al. found that ovarian morphology can reflect the degree of metabolic and reproductive derangements in patients with PCOS [24]. The count of follicles under 6 mm and AFC was positively associated with features of endogenous androgen excess. The OV, however, was not linked with any reproductive markers but showed negative associations with glycated hemoglobin (HbA1c) [24].

As technologies developed faster and new ultrasound machines with higher resolution transducers became available in most healthcare units, the guideline-creating groups from Europe, Australia, and America decided to alter the AFC from 12 to 20 or more follicles per ovary [12]. High-frequency transducers allow more precise images and thus should help distinguish more cysts. However, the new threshold appeared disadvantageous in the recent article by Kim et al. [38]. Their study revealed that women, who could be diagnosed with PCOS according to the previous cut-off, are now excluded from the diagnosis, even though they have irregular menstruation or symptoms of hyperandrogenism. Moreover, they discovered that the excluded women (the “low AFC group” who had 12–19 follicles per ovary) tended to have worse hormonal profiles, higher prevalence of MetS, and higher androgen levels in the blood than the control group [38].

3D ultrasonography is a novel perspective tool for diagnosing PCOS. It allows precise visualization of the ovarian morphology and may help establish a more accurate diagnosis [41]. Nylander et al. recently conducted a study in which AFC and OV were obtained using 2D and 3D transvaginal ultrasound and compared with MRI as the gold standard. 2D transvaginal ultrasound showed lower AFC and OV values than the 3D and MRI in the overweight population with PCOS. Furthermore, serum anti-Müllerian hormone, a biochemical marker of PCO, had a higher correlation with AFC, obtained from 3D, than from 2D transvaginal ultrasound [42]. In the study by Bozkurt et al., PCOS patients and women with multifollicular ovaries were evaluated using 2D and 3D ultrasonography [15]. The 2D Doppler measured the RI and PI; these parameters were slightly higher in the PCOS group, but the difference from the multifollicular ovary group was not statistically significant. However, the 3D Doppler measurements (vascularization index, flow index, and vascularization flow index) showed significantly higher values in the PCOS group. The authors suggest that 3D ultrasound with power Doppler would be an essential tool in differentiating PCOS and multifollicular ovaries [15]. However, some authors doubt the 3D ultrasound superiority over the 2D ultrasound in diagnosing PCOS. Sujata et al. discovered no significant differences between these ultrasound types in assessing ovarian morphology [43]. As the study findings remain controversial, more research is required to evaluate the effectiveness of the 3D ultrasound in establishing the PCOS diagnosis.

## 6 Conclusion

OV and FNPO are reliable markers of PCOS. However, the peripheral distribution of follicles is particular for PCOS. Higher ovarian stromal echogenicity might predict reproductive dysfunction. The Doppler ultrasound is a promising tool for evaluating the ovarian stromal artery; nevertheless, the value of assessing the uterine artery remains controversial.
